# The effect of variation in interpretation of the La Trobe radiographic foot atlas on the prevalence of foot osteoarthritis in older women: the Chingford general population cohort

**DOI:** 10.1186/s13047-017-0239-9

**Published:** 2017-12-08

**Authors:** Peter McQueen, Lucy Gates, Michelle Marshall, Michael Doherty, Nigel Arden, Catherine Bowen

**Affiliations:** 1 Faculty of Health Sciences, University of Southampton, Highfield Campus Building 45, Southampton, SO17 1BJ UK; 2 Arthritis Research UK Centre for Sport, Exercise and Osteoarthritis, University of Southampton, Southampton, UK; 3 Arthritis Research UK Centre for Sport, Exercise and Osteoarthritis, University of Oxford, Oxford, UK; 4 Arthritis Research UK Primary Care Centre, Research Institute for Primary Care & Health Sciences, Keele University, Keele, UK; 5 Arthritis Research UK Pain Centre and Academic Rheumatology, School of Medicine, University of Nottingham, Nottingham, UK; 6 Medical Research Council Lifecourse Epidemiology Unit, University of Southampton, Southampton, UK

**Keywords:** Foot, Feet, Joints, Osteoarthritis, Arthritis, Prevalence, Diagnosis

## Abstract

**Background:**

The prevalence of foot osteoarthritis (OA) is much less understood than hip, knee and hand OA. The foot is anatomically complex and different researchers have investigated different joints with lack of methodological standardisation across studies. The La Trobe Foot Atlas (LFA) is the first to address these issues in providing quantitative assessment of radiographic foot OA, but has not been tested externally. The aim of this study was to evaluate three different interpretive approaches to using the LFA for grading OA when scoring is difficult due to indistinct views of interosseous space and joint contour.

**Methods:**

Foot radiographs of all remaining participants (*n* = 218) assessed in the Chingford Women Study 23 year visit (mean (SD) for age: 75.5 years (5.1)) were scored using the LFA defined protocol (Technique 1). Two revised scoring strategies were applied to the radiographs in addition to the standard LFA analyses. Technique 2 categorised joints that were difficult to grade as ‘missing’. Technique 3 included joints that were difficult to grade as an over estimated score. Radiographic OA prevalence was defined for the foot both collectively and separately for individual joints.

**Results:**

When radiographs were scored using the LFA (Technique 1), radiographic foot OA was present in 89.9%. For Technique 2 the presence of radiographic foot OA was 83.5% and for Technique 3 it was 97.2%. At the individual joint level, using Technique 1, the presence of radiographic foot OA was higher with a wider range (18.3–74.3%) than Technique 2 (17.9–46.3%) and lower with a wider range (18.3–74.3%) than Technique 3 (39.9–79.4%).

**Conclusion:**

The three different ways of interpreting the LFA scoring system when grading of individual joints is technically difficult and result in very different estimates of foot OA prevalence at both the individual joint and global foot level. Agreement on the best strategy is required to improve comparability between studies.

## Background

Osteoarthritis (OA) is an important cause of global disability, with adult prevalence rates reported between 8.5–22.0% for symptomatic radiographic knee OA [1–3], 3.4–8.9% for symptomatic radiographic hip OA [2, 4, 5]. The prevalence of radiographic hand OA has been reported to range from 27.0 to 83.0% [2, 6]. Hand OA is said to consist of several phenotypes that make it more complex to study [7]. Whilst investigation of foot joints may be more aligned to those of the hand as a peripheral joint site with multiple small bones and joints, the prevalence of radiographic foot OA is much less understood.

Foot pain is often linked to foot OA and is highly prevalent in the general population, with estimates that range between 15.0–63.0% [8–11]. Although conventional radiographs have been used traditionally to assess OA there is discordance in how radiographic and symptomatic OA are defined [12–14] and a lack of methodological standardisation across studies [9]. For investigations of foot OA, issues such as the considerable variation in study populations, the radiographic views taken, which foot joints are examined, the grading systems applied and definitions for prevalence of radiographic foot OA are highlighted as potential reasons for the lack of conclusive data regarding radiographic and symptomatic foot OA [15]. Of these factors the lack of standardisation in the methods used to assess radiographic foot OA [15], the number of foot joints included to define foot OA [16] and the disparity between radiographic OA and symptomatic OA [17, 18] appear to be key issues to address. Recently, the UK population prevalence of symptomatic radiographic foot OA has been estimated as 16.7% in adults aged over 50 years [19] and in the US prevalence estimates of pain at specific foot locations range between 7 and 13% in adults (30–100 years) [20].

Experts agree that the separate grading of osteophytes (OPs) and joint space narrowing (JSN) using standardised and validated atlases is an important way forward [21, 22]. In an attempt to address this, Menz et al. [23] developed a radiographic atlas specifically for standardising the documentation and interpretation of foot OA. The atlas uses an ordinal scale to score the presence of OP and JSN at five joints within the foot on dorsoplantar and lateral views [23]. Previously investigators largely relied on the Kellgren and Lawrence classification system [24] to define OA in individual foot joints, which was often limited just to the first metatarsophalangeal joint (1stMTPJ) [12, 15, 25].

Menz et al. reported good intra-rater reliability (percentage agreement from 86.0 to 99.0% and weighted κ from 0.45 to 0.95), of the La Trobe Foot Atlas (LFA) and construct validity relative to foot symptoms [16, 23]. The LFA has since been used to determine the prevalence of radiographic OA at the global foot level in relation to foot pain [19] and effects of intervention at an individual joint level [26]. Studies using the LFA that did not include a member of the original team that developed the atlas are scarce [25] or do not discuss the use of the atlas [27] such that the interpretation of the LFA scoring has yet to be evaluated.

The presentation of radiographic features varies quite widely. As radiographic atlases use semi-quantitative or ordinal grading systems to classify individuals, often into 4 or 5 categories, a degree of interpretation is required in order to categorise OA features [28–31]. We postulated that, as with other radiographic atlases, the LFA ordinal technique for scoring introduces an interpretative approach, that may potentially lead to an over or under-estimation in the prevalence of OA [31]. This is particularly likely when an unclear view of a joint is being assessed, which happens often for views of the midfoot and certain hind-foot joints [16, 23, 32]. The authors of the original LFA themselves do suggest from their inter-rater reliability results that “there is some degree of inherent variability in the interpretation of some aspects of the atlas” [23]. We wished to evaluate how much this variation in interpretation can affect the prevalence of radiographic foot OA.

## Methods

### Study participants

**Fig. 1:**
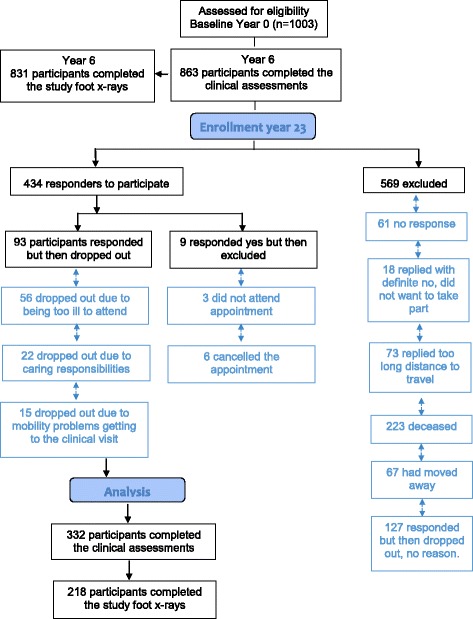
Participant recruitment flow diagram for the year 23 Chingford 1000 women’s study

Foot radiographs were sourced from a well-established population-based cohort of middle aged women - ‘The Chingford 1000 Women Study’ (http://www.chingfordstudy.org.uk) (see Fig. 1 recruitment flow chart). This prospective cohort originally comprised 1003 women aged 45–64 years from a general practice in Chingford, North-East London, UK. Participants have been followed annually since 1989 and are representative of women in the UK general population with respect to weight, height, and smoking characteristics, the details of which have been previously published [33–36]. The ‘Chingford 1000 Women Study’ has focussed on the natural history of OA and osteoporosis and has followed strict well-established protocols.

Full ethical approval was granted by Waltham Forest and Redbridge Local Research Ethics Committee (reference number: LREC R & WF 96). The study was sponsored by Whipps Cross Hospital Research and Development Unit. An amendment application was approved for the year 23 clinical foot assessment study by NRES Committee South Central – Oxford A (May 2013; REC number: 84,131). All participants gave fully informed written consent.

### Foot radiographs

A sample of foot radiographs taken at year 23 (collected between 2013 and 2014), were used to evaluate prevalence of radiographic foot OA between different scoring techniques. The sample included all women who returned for the 23 year visit and had foot radiographs taken (*n* = 218) with mean (SD) age: 75.5 years (5.1); weight: 69.2 kg (12.6); height: 158.4 cm (6.1); BMI: 27.6 (4.8). The participants at the year 23 visit were therefore older with a higher BMI than at the baseline year 0 visit, mean (SD) age: 54.1 (6.4) and BMI: 25.3 (4.3). There was no significant difference in age or BMI between those who attended for foot x-ray and those who did not at year 23.

All radiographs at this time point were taken weight bearing in two views (dorsoplantar and lateral) of each foot according to the LFA defined protocol [23, 37] and stored on disc.

### Radiographic scoring of foot osteoarthritis

The LFA focuses on 5 of the 32 joints of each foot, specifically: the 1^st^ MTPJ; the first cuneiform-metatarsal joint (1^st^CMJ); the second cuneiform-metatarsal joint (2ndCMJ); the navicular-first cuneiform joint (N1stCJ); and the talo-navicular joint (TNJ). A four-point scale of 0, 1, 2 and 3 is used to score OPs (0 = absent; 1 = small; 2 = moderate; 3 = severe) and JSN (0 = none; 1 = definite; 2 = severe; 3 = bone-on-bone at least one point) in both feet in both the dorsoplantar and lateral view. Although the scale description proposed in the LFA publication [23] describes JSN grade 3 as “joint fusion” we have interpreted this more precisely as “bone-on-bone”. Foot OA is considered to be present if a score of 2 or more is documented for either OP or JSN on either of the two views [23].

Pictorial guidance for each grade of OP and JSN is provided for each view. The exception is the TNJ for OP grading on the dorsoplantar view, which was excluded from the LFA as the authors asserted that OP more commonly develops on the dorsal aspect of this joint which is difficult to visualize from a dorsoplantar projection [23]. Joints that could not be scored (e.g. Due to surgical removal or presence of other pathology) were excluded from the analyses.

All radiographs were scored by a single trained reader (PMc).

#### Scoring techniques

Two revised scoring methods were applied to the foot radiographs in addition to the standard LFA analyses to determine prevalence of radiographic foot OA between different interpretive approaches:

- **Technique 1** was employed as the LFA standard technique [23] whereby all joints that were difficult to interpret and score for OPs and/or JSN were given a score based on a conservative estimate. (For example, where an OP in a participant’s joint may have been ambiguous to score between a grading of 2 or 3, the lower grading of ‘2’ was accepted).
- **Technique 2** was a new approach not used previously. It was devised by our team of experts in the field of osteoarthritis (NKA, MD), radiography (MM) and foot and ankle research (CB) to understand how the prevalence estimate of foot OA changed when a scorer did not include an estimate for any joint they could not make a decision on.
  All joints that were difficult to interpret and score for OPs and/or JSN were designated as missing values and excluded from the analyses (under-estimate).
- **Technique 3** was a revised version of Technique 1 whereby all joints that were difficult to interpret and score for OPs and/or JSN were given a score based on an over-estimate. (For example, where an OP in a participant’s joint may have been ambiguous to score between a grading of 2 or 3, the higher grading of ‘3’ was accepted).

#### Scorer reliability

The reader (PMc) had undergone training by an experienced radiographer (MM) who had used the LFA in a previous OA foot study [19, 37]. Using a sample (*n* = 20) of archived radiographs of both feet (Chingford year 6) and the LFA standard technique (Technique 1), for dorsoplantar views, intra-rater agreement was established for each LFA category at the individual joint level by overall percentage agreement and weighted kappa statistics (for categorical scoring) of OPs and JSN) based on value criteria by Landis and Koch [38]. For the five joints and both feet the range was fair to substantial for OPs and poor to substantial for JSN; percentage close agreement ranged from 47.6–85.7% for OPs and from 33.3–81.0% for JSN (Table 1).

**Table 1:** Intra-rater agreement for ordinal radiographic feature scores (0–3) in individual joints

	Joints	Osteophytes	Joint space narrowing
Left foot (n = 20) Dorsoplantar view	1st Metatarsophalangeal joint	Kw = 0.5; pca = 53%	Kw = 0.6; pca = 81%
	1^st^ Cuneo-metatarsal joint	Kw = 0.4; pca = 48%	Kw = 0.5; pca = 57%
	2nd Cuneo-metatarsal joint	Kw = 0.8; pca = 86%	Kw = 0.7; pca = 81%
	Navicular 1st cuneiform joint	Kw = 0.5; pca = 67%	Kw = 0.6; pca = 76%
	Talo-navicular joint	^a^	Kw = 0.2; pca = 67%
Right foot (n = 20) Dorsoplantar view	1st Metatarsophalangeal joint	Kw = 0.6; pca = 62%	Kw = −0.1; pca = 33%
	1st Cuneo-metatarsal joint	Kw = 0.5; pca = 57%	Kw = 0.5; pca = 62%
	2nd Cuneo-metatarsal joint	Kw = 0.3; pca = 79%	Kw = 0.5; pca = 67%
	Navicular 1st cuneiform joint	Kw = 0.6; pca = 86%	Kw = 0.6; pca = 71%
	Talo-navicular joint^a^	^a^	Kw = 0.3; pca = 71%

Key: *Kw* Stata pre-recorded weighting was used for kappa weighting disagreements, *pca* percentage agreement was based on all LFA categories (0–3)

^a^ Osteophyte scores for the talonavicular joints were not computed as this is not included within the AFA guidance

### Statistics

Data evaluation and statistical analyses were performed using Stata version 13.0 (Stata Corp, College Station, Texas, USA). The distribution of data was initially examined using histograms and scatter plots. No ‘outliers’ were found that may have occurred due to data entry bias or normal biological outliers. Assessment of the different radiographic scoring techniques are described using frequency (%) of radiographic foot OA at person foot level and individual joint level. Differences between the techniques are reported as frequency range.

## Results

When the foot radiographs (Chingford year 23) were scored using the LFA (Technique 1), the total (i.e. combined joints of left and right feet) prevalence of radiographic foot OA in any joint in the right and left foot was 81.2% using only the dorsoplantar view and 83.5% using only the lateral view. When scores were combined for both views and both feet radiographic foot OA was present in 89.9% of participants (Table 2). For Technique 2 (categorising joints that were difficult to grade as ‘missing’) the prevalence of radiographic foot OA was 83.5% (both feet, both views). For Technique 3 (attributing an over estimated score to joints that were difficult to grade) the prevalence of radiographic foot OA was 97.2% (Table 3).

**Table 2:** Frequency of radiographic foot OA according to Technique 1 and Technique 2 scoring methods

Foot	Joints	Radiographic view	Technique 1%OA^a^ (n)	Number of ungradable joints^b^	Technique 2%OA^a^ (n)	Technique 1–2 Difference %OA	Total number of participants
Left	1st MTPJ	Dorsoplantar	27.1 (59)	0	27.1 (59)	0.0	218
		Lateral	22.9 (50)	21	13.7 (27)	7.2	197
		Combined	35.8 (78)	0	31.2 (68)	4.6	218
	1st CMJ	Dorsoplantar	45.0 (98)	5	43.2 (92)	1.8	213
		Lateral	10.1 (22)	5	8.5 (18)	1.6	213
		Combined	49.1 (107)	0	45.9 (100)	3.2	218
	2nd CMJ	Dorsoplantar	49.5 (108)	74	30.6 (44)	18.9	144
		Lateral	57.3 (125)	75	27.3 (39)	30.0	143
		Combined	74.3 (162)	21	38.1 (75)	36.2	197
	N1stCJ	Dorsoplantar	19.3 (42)	3	18.1 (39)	1.2	215
		Lateral	11.0 (24)	35	8.7 (16)	2.3	183
		Combined	24.3 (53)	1	21.2 (46)	3.1	217
	TNJ	Dorsoplantar	7.3 (16)	0	7.3 (16)	0.0	218
		Lateral	21.1 (46)	1	19.8 (43)	1.3	217
		Combined	24.3 (53)	0	22.9 (50)	1.4	218
Right	1st MTPJ	Dorsoplantar	33.0 (72)	1	32.7 (71)	0.3	217
		Lateral	27.5 (60)	25	16.1 (31)	11.4	193
		Combined	42.2 (92)	1	35.9 (78)	6.3	217
	1^st^ CMJ	Dorsoplantar	47.2 (103)	3	46.0 (99)	1.2	215
		Lateral	9.2 (20)	3	7.4 (16)	1.8	215
		Combined	49.5 (108)	0	46.3 (101)	3.2	218
	2nd CMJ	Dorsoplantar	47.7 (104)	80	29.7 (41)	18.0	138
		Lateral	56.4 (123)	76	22.5 (32)	33.9	142
		Combined	70.6 (154)	29	34.4 (65)	36.2	189
	N1stCJ	Dorsoplantar	17.9 (39)	3	16.7 (36)	1.2	215
		Lateral	8.7 (19)	35	6.6 (12)	2.1	183
		Combined	22.5 (49)	2	20.4 (44)	2.1	216
	TNJ	Dorsoplantar	7.8 (17)	0	7.8 (17)	0.0	218
		Lateral	15.1 (33)	1	14.7 (32)	0.4	217
		Combined	18.3 (40)	0	17.9 (39)	0.4	218
Both	ALL 5 joints	Dorsoplantar	81.2 (177)	0	78.4 (171)	2.8	218
		Lateral	83.5 (182)	0	57.3 (125)	26.2	218
		Combined	89.9 (196)	0	83.5 (182)	6.4	218

Key: *1st MTPJ* first metatarsophalangeal joint, *1* ^*st*^ *CMJ* the first cuneiform-metatarsal joint, *2nd CMJ* the second cuneiform-metatarsal joint, *N1stCJ* the navicular-first cuneiform joint, *TNJ* talo-navicular joint

^a^Positive diagnosis of radiographic osteoarthritis (AFA grade ≥ 2)

^b^ to be counted as an ‘ungradable’ joint issues with the scoring of both OP and JSN features in the view being examined had been documented

**Table 3:** Frequency of radiographic foot OA according to Technique 1 and Technique 3 scoring methods

Foot	Joints	Radiographic view	Technique 1%OA* (n)	Technique 3%OA* (n)	Technique 1–3 Difference %OA	Total number of participants
Left	1st MTPJ	Dorsoplantar	27.1 (59)	38.1 (83)	- 11.0	218
		Lateral	22.9 (50)	23.4 (51)	- 0.5	197
		Combined	35.8 (78)	42.7 (93)	- 6.9	218
	1^st^ CMJ	Dorsoplantar	45.0 (98)	61.9 (135)	- 16.9	213
		Lateral	10.1 (22)	15.6 (34)	- 5.5	213
		Combined	49.1 (107)	65.1 (142)	- 16.0	218
	2nd CMJ	Dorsoplantar	49.5 (108)	55.5 (121)	- 6.0	144
		Lateral	57.3 (125)	64.2 (140)	- 6.9	143
		Combined	74.3 (162)	79.4 (173)	- 5.1	197
	N1stCJ	Dorsoplantar	19.3 (42)	69.3 (151)	- 50.0	215
		Lateral	11.0 (24)	16.1 (35)	- 5.1	183
		Combined	24.3 (53)	73.4 (160)	- 49.1	217
	TNJ	Dorsoplantar	7.3 (16)	22.9 (50)	- 15.6	218
		Lateral	21.1 (46)	30.7 (67)	- 9.6	217
		Combined	24.3 (53)	43.6 (95)	- 19.3	218
Right	1st MTPJ	Dorsoplantar	33.0 (72)	46.3 (101)	- 13.3	217
		Lateral	27.5 (60)	28.9 (63)	- 1.4	193
		Combined	42.2 (92)	52.3 (114)	- 10.1	217
	1^st^ CMJ	Dorsoplantar	47.2 (103)	63.8 (139)	- 6.6	215
		Lateral	9.2 (20)	14.2 (31)	- 5.0	215
		Combined	49.5 (108)	66.5 (145)	- 17.0	218
	2nd CMJ	Dorsoplantar	47.7 (104)	55.5 (121)	- 7.8	138
		Lateral	56.4 (123)	60.1 (131)	- 3.7	142
		Combined	70.6 (154)	74.8 (163)	- 4.2	189
	N1stCJ	Dorsoplantar	17.9 (39)	72.5 (158)	- 54.6	215
		Lateral	8.7 (19)	12.8 (28)	- 4.1	183
		Combined	22.5 (49)	74.8 (163)	- 52.3	216
	TNJ	Dorsoplantar	7.8 (17)	25.2 (55)	- 17.4	218
		Lateral	15.1 (33)	26.1 (57)	- 11.0	217
		Combined	18.3 (40)	39.9 (87)	- 21.6	218
Both	ALL 5 joints	Dorsoplantar	81.2 (177)	92.7 (202)	- 11.5	218
		Lateral	83.5 (182)	89.9 (196)	- 6.4	218
		Combined	89.9 (196)	97.2 (212)	- 7.3	218

Key: *1st MTPJ* first metatarsophalangeal joint, *1* ^*st*^ *CMJ* the first cuneiform-metatarsal joint, *2nd CMJ* the second cuneiform-metatarsal joint, *N1* ^*st*^ *CJ* the navicular-first cuneiform joint, *TNJ* talo-navicular joint

*Positive diagnosis of radiographic osteoarthritis (AFA grade ≥ 2)

At the individual joint level, Technique 2 elicited a lower presence of radiographic foot OA than Technique 1 (Table 2). With the exception of the 2^nd^ CMJ (both feet and both views) that elicited a difference of 36.2% (both feet) between Techniques 1 and 2 joint scores, all other joint scores were within an acceptable range (left foot: 1.4–4.6%; right foot: 0.4–6.3%). Conversely, at the individual joint level Technique 3 elicited a higher presence of radiographic foot OA than Technique 1. With the exception of the N1stCJ (both feet, dorsoplantar view) that elicited a difference of 49.1% (left foot) and 52.3% (right foot) between Technique 1 and 3 scores, all other joint scores were within a less wide range (left foot: 5.1–19.3%; right foot: 4.2–21.6%).

At the individual joint level, using Technique 1, the presence of radiographic foot OA for combined OP and JSN was higher with a wider range (18.3–74.3%) than Technique 2 (17.9–46.3%). At the individual joint level, using Technique 1, the presence of radiographic foot OA for combined OP and JSN was lower with a wider range (18.3–74.3%) than Technique 3 (39.9–79.4%).

## Discussion

In this study, we sought to extend knowledge of radiographic foot OA by examining three different interpretive approaches to classifying foot OA using the LFA. The three different ways of interpreting the LFA scoring system when scoring individual joints that we used is technically difficult and each resulted in different estimates of foot OA prevalence at both the individual joint and global foot level.

Similar to other radiographic scoring methods, such as Kellgren and Lawrence [24, 39] and the Osteoarthritis Research Society International (OARSI) atlas [40], there is potential ambiguity in the interpretation of the scoring for OPs and JSN within individual joints using the LFA. Scoring of foot joints on radiographs presents specific problems due to overlap of bones that makes it difficult to clearly see the joint line and OP on any one view in all joints of interest. Through comparison of the different techniques we showed the potential for the range of prevalence estimates of person level radiographic foot OA to be between 83.5% and 97.2%.

Menz et al. [16] reported the prevalence of radiographic foot OA in their elderly sample (as 93%, which is similar to our standard LFA assessment of 89.9% and within our range when utilising the two additional techniques. Menz et al. [16] also reported a joint-specific prevalence rate for individual joints that ranged between 23.0–60.0% which is similar to the range between 18.3–74.3% that we found. The sample size that Menz et al. [16] investigated and age was similar to ours (*n* = 197, mean age 75.9 years, [SD] 6.6), however they were drawn from a retirement village and a university health sciences clinic in Melbourne, Victoria, Australia with 64.0% women, whilst ours were all women drawn from a general population in the UK.

Other investigators have reported lower prevalence estimates for foot OA. For example, in an American population, the Clearwater Osteoarthritis Study, a prospective cohort consisting of 3463 participants (40–94 years), Wilder et al. [41] reported a prevalence of 20.0% of radiographic foot OA. Within that study, the focus was on one only foot joint only, the 1st MTPJ, so a lower prevalence of OA at the individual foot joint level is expected. Our findings were higher 35.8% (left) and 42.2% (right) for presence of OA in the 1st MTPJ. The lower estimate produced by Wilder et al. [41] may be due to the fact that their scoring was based on the traditional Kellgren and Lawrence scale which is not as sensitive to radiographic foot OA as the LFA [16].

It is not just the approach that is open to interpretation. Even using the different techniques, our estimates are much higher, than the most recent UK study that estimated the population prevalence of symptomatic radiographic foot OA as 16.7% [19]. The latter study used foot pain and foot OA (ie symptoms plus radiographs) to define their prevalence of symptomatic foot OA, whereas we only used foot OA (radiographs). This highlights the marked difference in prevalence estimates dependent on whether the focus of investigation is on symptomatic radiographic foot OA or just radiographic foot OA, the latter being distinctly much higher [2]. The difference in prevalence estimates due to the case definition has been noted in OA at other joints sites [2].

Each of these examples may go some way to explaining the variation in published prevalence estimates of radiographic foot OA, especially when different techniques are employed and different joints included. Other factors that may explain the differences in prevalence estimates of radiographic foot OA could be related to the subjectivity of the scoring method being ordinal as opposed to objective measurements such as joint space width. As a further example, we found that scoring may be confounded as the individual features of OP or JSN are not presented separately but are mixed and this may distract the scorer to judge the “best-fit” picture due to the overall appearance rather than to just the OP or JSN they are scoring.

The advice given in the LFA indicates that use of both dorsoplantar and lateral views is ‘gold standard’ and should be applied where possible to ensure an appropriate level of sensitivity to OA [23]. Further evaluation of the LFA has shown that good sensitivity (94.6%) can be obtained in the 1st MTPJ when only a dorsoplantar view is available. However, substantially lower sensitivity was achieved for the other joints (between 31.0 and 60.7% of cases) [16]. The 1^st^ MTPJ is the largest of the MTPJs and is not obscured by other joints when observed in radiographs and as such easier to assess the presence of OPs and JSN. Menz et al. [16] reported the combined view was 42.4% for the 1^st^MTPJ which is very similar to our estimate of combined view 1^st^ MTPJ presence of OA as 35.8% for the left foot (dorsoplantar view: 27.1%; lateral view: 22.9%) and 42.2% for the right foot (dorsoplantar view: 33.0%; lateral view: 27.5%). Of note, the joints that showed most difference between our techniques were the 2nd CMJ and N1stCJ. These joints are also the ones noted to be difficult to score in the LFA atlas due to considerable amount of overlap of bones and joints [23].

There are limitations to this study. Firstly, it is possible that our estimates of prevalence may have been confounded by the lower reproducibility of the rater in this study than that of the original authors of the LFA [23]. There are a number of explanations for lower reliability scores in our preliminary work such as the foot positioning for the reliability study differed from that undertaken by the LFA, availability of only one view (non-weight-bearing dorso-plantar) and lower quality of foot radiographs versus higher quality of resolution of electronic images used in year 23. For the development and testing of the LFA, the same authors selected the radiographs for each LFA classification grade on which their reliability was calculated [23]. This may provide more stable predictions of reliability scores but may not be as readily applicable to new raters external to the original development team.

Secondly, the cohort used in the development of the LFA was a sample of the Australian population over the age of 65 years, whereas the foot radiographs used in this study were all from a sample of women of the UK population aged 69–93 years. There is currently no available foot radiographic data that compares different populations that may have different physiology, anatomy and genetics. Consequently, we do not know how representative the pictures used to explain the scoring method within the LFA are for global comparisons or how closely the Chingford 1000 Women’s study cohort foot radiographs may align to them. Of note, within the LFA there are not separate pictures for OPs or JSN for men and women. It is currently not known if factors such as joint width are smaller in foot joints of women than men which may affect interpretation and scoring for OA.

Thirdly, differences in prevalence estimates of foot OA could be related to study populations and the focus of the investigation. The focus of our investigation was radiographic features of foot OA only. We have not aligned this to symptoms of foot pain as our aim was to evaluate the scoring technique for foot OA using a validated radiographic atlas. Our findings are therefore not directly comparable with other investigators reporting on the prevalence of symptomatic foot OA. Whilst symptomatic foot OA may be more prevalent in women [19] we are aware that the prevalence of foot OA was very high in our study. We believe this could be due to a combination of the population being all women aged over 69 years in whom OA has generally been found to be more prevalent [42]. Additionally, in our study, OA was defined radiographically which has been shown to lead to higher estimates than other definitions such as ‘self-reported OA’ and ‘symptomatic OA’ (combined radiographic OA with symptoms) [2]. Estimates of the prevalence of OA of a similar order have been reported at other peripheral joints sites in other populations of older women [43].

## Conclusion

This study supports the use of the La Trobe Foot Atlas to facilitate standardised scoring of foot OA in existing current and historical radiographs of established large population cohorts with the caveat that the interpretative scoring technique requires acknowledgment. We have evaluated three different ways of interpreting the scoring system when scoring of individual joints is technically difficult and results in different estimates of foot OA prevalence at both the individual joint and global foot level. This strengthens the case for further refinement of definitions for foot OA between investigators and improved comparability between studies. Future work should focus on agreement on the best strategy to improve comparability between studies to begin to identify the risk factors for foot OA. From that the field can move forward in developing best clinical strategies for prevention and management of foot OA.

## Abbreviations

LFA: The La Trobe Foot Atlas
OA: Osteoarthritis
